# Cells in Dengue Virus Infection In Vivo

**DOI:** 10.1155/2010/164878

**Published:** 2010-08-12

**Authors:** Sansanee Noisakran, Nattawat Onlamoon, Pucharee Songprakhon, Hui-Mien Hsiao, Kulkanya Chokephaibulkit, Guey Chuen Perng

**Affiliations:** ^1^Department of Pathology and Laboratory Medicine, Dental School Building, Emory Vaccine Center, Emory University School of Medicine, 1462 Clifton Road, Atlanta, GA 30322, USA; ^2^Medical Biotechnology Unit, National Center for Genetic Engineering and Biotechnology, National Science and Technology Development Agency, Pathumthani 12120, Thailand; ^3^Office for Research and Development, Faculty of Medicine Siriraj Hospital, Mahidol University, Bangkok 10700, Thailand; ^4^Department of Pediatrics, Siriraj Hospital, Mahidol University, Bangkok 10700, Thailand

## Abstract

Dengue has been recognized as one of the most important vector-borne emerging infectious diseases globally. Though dengue normally causes a self-limiting infection, some patients may develop a life-threatening illness, dengue hemorrhagic fever (DHF)/dengue shock syndrome (DSS). The reason why DHF/DSS occurs in certain individuals is unclear. Studies in the endemic regions suggest that the preexisting antibodies are a risk factor for DHF/DSS. Viremia and thrombocytopenia are the key clinical features of dengue virus infection in patients. The amounts of virus circulating in patients are highly correlated with severe dengue disease, DHF/DSS. Also, the disturbance, mainly a transient depression, of hematological cells is a critical clinical finding in acute dengue patients. However, the cells responsible for the dengue viremia are unresolved in spite of the intensive efforts been made. Dengue virus appears to replicate and proliferate in many adapted cell lines, but these in vitro properties are extremely difficult to be reproduced in primary cells or in vivo. This paper summarizes reports on the permissive cells in vitro and in vivo and suggests a hematological cell lineage for dengue virus infection in vivo, with the hope that a new focus will shed light on further understanding of the complexities of dengue disease.

## 1. Introduction

Dengue is one of the most important mosquito-borne viral diseases affecting humans, with over half of the world's population living in areas at risk. Originally, dengue virus infections occurred mainly as epidemics in tropical and subtropical countries. But over time, with increasing globalization and the geographic spread of inhabitants of *Aedes aegyti* and *Aedes albopictus* mosquitoes, the dominant vectors for dengue virus transmission, dengue virus infection has steadily penetrated every corner of the world [1, 2]. Dengue virus has four serotypes, and each of them can cause a spectrum of diseases ranging from asymptomatic, mild febrile (dengue fever, DF) to a life-threatening illness, dengue hemorrhagic fever (DHF)/dengue shock syndrome (DSS). Approximately 50 to 100 million people contract dengue fever annually, and about 200,000 to 500,000 of these are DHF/DSS, which has a mortality rate about 1%–5%, mainly in children under 15 years of age [3]. 

Clinically, DF and DHF/DSS have several common features: viremia lasting for 5 to 8 days, fever persisting for 2 to 7 days, headache, myalgia, bone/joint pain, and rash, often accompanied by leucopenia. Occasionally variable degrees of thrombocytopenia and cutaneous hemorrhage are observed. More severe cases with incapacitating bone/joint pain (“break-bone-fever”) are common among adults. The pathological hallmarks that determine disease severity and distinguish DHF from DF and other viral hemorrhagic fevers are plasma/vascular leakage resulting from increased vascular permeability and abnormal hemostasis. Factors and biomarkers that can be used to identify those individuals at risk for DHF/DSS are not known. Epidemiological evidence suggests that preexisting immunity to dengue virus can enhance disease upon sequential infections [4]. Although intense efforts have been made to identify the etiology of DHF/DSS, the potential mechanisms involved in the pathogenesis of DHF/DSS remain an enigma; in large part due to the lack of a satisfactory animal model that recapitulates the clinical sequelae of human dengue virus infection. Currently, there are no effective vaccines or therapeutic drugs available to prevent or treat dengue virus infection. The importance of the dengue, in particular the more severe and potential dire consequences including death in DHF/DSS, has caught the attention of public concerns, and the NIAID/NIH has listed dengue virus as a Category A priority biothreat pathogen [5]. The recent outbreak in Brazil highlights the possibility of dengue virus spread to North Americas, thus providing a potential public health threat to the US as outlined by Dr. Fauci, NIAID [6]. 

Dengue is a timing illness, in other words, the progression to clinical manifestations may differ among infected individuals, which has caused variation in time points of specimen sampling. Currently, many of the descriptive events or associated factors related to dengue or dengue pathogenesis are predominantly derived from the specimens obtained at the appearance of clinical signs of dengue. Because of the lack of early time point in patient samples and suitable or satisfactory animal models, a comprehensive picture of the events cumulating in DHF/DSS pathogenesis, such as the role of enhancing antibodies, the requirement for specific sequence of infection, the types of cells infected, as well as the nature and source of the mediators responsible for increased vascular permeability, is unresolved and constantly in debate. 

In this paper, we summarize or discuss what has been reported thus far on the permissive cells for dengue virus infection both in vitro and in vivo and propose a new potential permissive cell type that has been neglected frequently and deserves much more attention.

## 2. Dengue Viruses

Dengue viruses, similar to other flaviviruses, possess a positive single-stranded RNA genome packaged inside a core protein, which is surrounded by an icosahedral scaffold and encapsidated by a lipid envelope. Its 11 kb genome functions similar to mRNA, encoding a polyprotein which upon translation is cleaved into three structural proteins (C, prM/M, and E) and seven nonstructural proteins (NS1, NS2A, NS2B, NS3, NS4A, NS4B, and NS5) by viral or host proteases. Since dengue viral genome can function as mRNA, if the viral RNA can be delivered into a cell's cytoplasm through biologically active vesicles, translation and genome synthesis can occur accordingly [7].

## 3. Dengue Viremia

Viremia is a common clinical manifestation in several severe viral infections. However, dengue viremia is unique because in endemic regions, where majority of the population has demonstrable neutralizing antibody to all four dengue serotypes [8], viremia still occurs in some of these populations upon bitten by mosquitoes carrying infectious dengue virus. The reasons why certain individuals developed clinical illness are not known, although an individual's genetic background, the interval between reinfection, sequence of infection by specific serotype, and quality of immune responses may partially account for the differences [4, 8]. Since identifying the permissive cell lineage(s) in vivo may uncover the underlying mechanisms leading to DHF/DSS and aid in vaccine and antiviral drug development, the source(s) of circulating virus in acute dengue patients has been the central focus for several decades. In spite of the efforts made to identify these cell(s), the question remains elusive.

## 4. In Vitro Studies

In vitro, numerous primary cell lineages and established cell lines have been studied for their relative permissiveness for dengue virus infection, including endothelial and fibroblast cells, myeloid-derived cells, and lymphocytes [9–17]. Although some of the cells defined in vitro could be permissive cells for dengue virus replication in vivo [18–21], the actual phenotypes of these cells have not been delineated or defined in detail. Consequently, conflicting reports abound in the literature. 

Historically, dengue virus has been isolated from polymorphonuclear leukocytes (PMNs) [22], adherent cells presumed to be phagocytic monocytes or macrophages [23], and nonadherent leukocytes [24, 25] from dengue patients. Additionally, since this virus is delivered to its host via mosquito bites to the skin, the human Langerhans cells, skin cells with a morphology and function similar to that of dendritic cells, have been suggested to be a potential target for dengue virus infection [26]. Several in vitro studies utilizing myeloid-derived dendritic cells have been documented, which suggest the permissive cells upon contact with dengue virus are monocytoid-derived DC-SIGN bearing DCs and mannose receptor bearing macrophages [27–33]. In this regard, however, other evidence suggests that Langerhans and/or dendritic cells are probably implementing their normal immune functions, such as taking up antigens for processing and presenting them to the adaptive immune cells, instead of serving as the reservoir cell for dengue virus [21, 34–36]. In addition, it should be noted that atypical lymphocytes, which may be cells closely related to CD19^+^ B cells, since there is a correlation between these two cell populations [37], have been regularly reported to be found in increasing frequency, circulating in the peripheral blood of naturally dengue-virus-infected human patients [38, 39]. This uncharacterized cell lineage has been suggested as a potential host cell for the replication of dengue virus in infected patients [22]. As a whole, only a small subpopulation of cells in peripheral blood appears to be infected by dengue virus [22, 23], but the phenotype of this subpopulation has yet to be fully characterized. A view on the selected suggestive permissive cells is elaborated in a bit more detail.

## 5. Skin Innate Immune Cells

**Figure 1:**
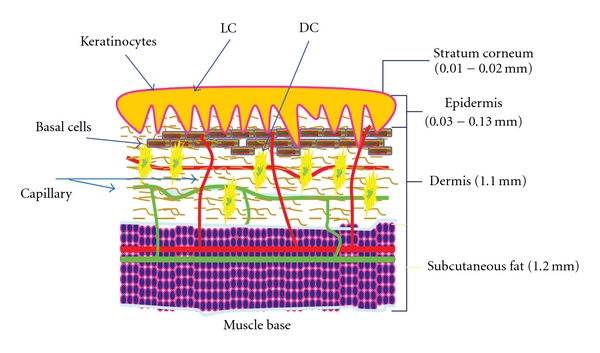
*A schematic diagram of the skin*. A cartoon drawing based upon the textbook descriptions of the thickness of outer skin layers. Only layers relevant to the subject are shown. LC, Langerhans cells; DC, dendritic cells; Capillary, green and red internetworks.

Dengue disease is introduced to its hosts by the bite of mosquitoes carrying infectious virus. The first obstacle that the mosquito encounters is the physical barrier of the skin, which is composed of several layers of keratinocytes interspersed with a network of capillaries (Figure 1). Keratinocytes are on the outermost epidermal layer of the skin, are endowed with Toll-like-receptors (TLR) [40], and may be considered a component of the primary innate immune system. Langerhans cells mainly reside in the thin layer of the epidermis, which does not contain capillaries, while dendritic cells are predominantly in the thicker dermis layer, which is filled with capillaries. Although Langerhans cells, in general, have the same phenotype as dendritic cells, and is impossible to distinguish activated Langerhans cells from dendritic cells by morphological appearance, numerous studies indicate that biological activities are discernible between these two cell types [41–44]. Many interesting questions can be asked. How does dengue virus interact with skin cells during mosquito probing prior to penetration? How deep does the mosquito fascicle penetrate into the skin? How does dengue virus behave upon contacting epidermal and dermal innate immune cells after the mosquito fascicle penetrates? And how does dengue virus get deposited and disseminated during the engorgement period while the mosquito imbibes the blood? The answers to these questions can elucidate how the fates of the cells on or in the skin are orchestrated.

## 6. Mosquito Imbibing

Gordon and Lumsden, the authors of a historical in vivo frog's web paper in 1939, observed that the mosquito's proboscis is flexible and predominantly imbibes blood directly from the capillary and only occasionally from the pools formed in the tissues by the leakage of blood from the capillary previously lacerated by the mosquito's proboscis [45]. This study is later confirmed in mice ear and human beings implementing the same experimental designs [46, 47]. The dimensions of an A*edes aegypti* fascicle are typically 1.8 mm in length with an internal radius of 10 *μ*m [48] and typically engorge a blood meal of 4.2 *μ*l in 141s [48]. It is estimated that during imbibing, approximately 50% (~0.9 mm) of the fascicle penetrates into skin [49], suggesting that the location of blood drawn from is the capillary-rich dermis layer, implicating that pathogens may be directly injected into the blood.

## 7. Dendritic and Langerhans Cells

Mosquito probing, penetration, and feeding on the surface of the skin is easily interrupted by the movement of the host. Unsuccessful imbibing may result in a certain amount of virus deposited on the outermost layers of skin, where keratinocytes, Langerhans, and dendritic cells may encounter the virus. The delicate alarm system of the skin can sense the probing of the mosquito and the penetration of the fascicle, potentially initiating a signaling cascade and the activation of defense mechanism. Thus, if these dendritic cells are permissive as others suggested [27–33], we would anticipate quite high incidence of the dengue cases in endemic regions during the rainy season. The critical role of these antigen presenting cells (APCs) is to ingest foreign particles including viruses, process these materials while migrating to the regional lymph nodes. Here, the APCs can present the foreign proteins to other immune cells, such as T cells, to initiate the cascade of the adaptive immune responses, including antibody production. Dendritic cells, therefore, may be more important for the induction of the host's defense. Importantly, it is of benefit to the host that the virus be engulfed and processed in order to generate an adequate immune response against the invading pathogen and protect the host from further infection. Since such phagocytic cells are the first line of defense in our body, this may perhaps explain why a majority of dengue cases are asymptomatic.

Interestingly, apoptotic keratinocytes and dendritic cells are observed in human skin explants when dengue virus is directly injected into the epidermis with a fine needle [35]. Furthermore, others have observed that mosquitoes can deposit high doses of virus extravascularly as they probe and feed on the host, while only a small amount of virus is injected directly into the blood [50]. Considering the fact that a majority of dengue virus infections are asymptomatic, this evidence suggests that the role of dendritic cells at the site of fascicle penetration is to eliminate or temporarily contain the intruders and thereby prevent or reduce the dissemination of dengue virus. However, the role of keratinocytes and dendritic cells in clearance of dengue virus remains to be further investigated.

## 8. Monocytes/Macrophages

Since dengue viral antigens are detectable in adherent cells obtained from the peripheral blood of dengue patients, monocytes and/or macrophages have been an assumptive target cell for more than three decades. With the high level of interest in the pathogenesis of DHF/DSS, intensive efforts have been made to identify the infected monocytes and/or macrophages in the peripheral blood of infected patients, and some suggestive successes have been documented. However, dengue is a timing disease. Specimens collected from dengue patients are often after the onset of clinical manifestations; therefore, the intervals prior to symptoms developed are different among individuals and are likely at the peak of dengue viremia, and autopsy samplings are always at the convalescent stage or later. Within the context, identifying a cell that is positive for dengue viral antigens in collected specimens requires meticulous investigations and cautious interpretations. Although recently researchers are attempting to address the issue with small animal models, such as the AG129 mice experimentally infected with dengue virus, the major pitfall of this model is that mice have a defective interferon response, which has been shown to play a very critical role in controlling virus replication and proliferation. Consequently, dengue viral RNA or antigens are observed in almost all the cells and organs that have been investigated [18, 21]. Within the same content, this same group investigated the autopsy tissues from patients who died of dengue virus infection. The authors showed that human tissues and the corresponding mice AG129 tissues were positive for dengue virus NS3 antigen, concluding that these cells propagated virus. However, the phenotypic markers of the cells that were positive for dengue viral antigen were not confirmed, and thus a conclusion was drawn based upon the similarities between humans and mice. Also, a new finding suggests that liver sinusoidal CD31+ endothelial cells in AG129 mice are positive for dengue viral antigen and can support the antibody-mediated infection [21]. However, evidence indicates that there are many differences in immunological and antiviral responses between humans and mice [51–53]. Thus, clarifications of the role of monocytes and macrophages in dengue virus infection in vivo are urgently needed. This notion is also applied to the paper published by Jessie et al. [20], in which the cell phenotype markers in those cells positive staining for either dengue viral antigens or RNA, were not confirmed. 

In addition, Durbin et al. [19] has performed an extensive phenotyping of PBMCs during acute dengue illness, and the results suggest that quite a few immune cells with various cell surface markers are positive for viral antigens, prM or NS3. Recently, in a study with AG129 mice, dengue antigens are seen in CD31^+^ liver cells stained with the same antibody [21]. However, these observations can be explained by several factors. One of such alternative explanation is platelet-leukocyte aggregation, which has been documented to occur in a number of physiological and pathological states [54–58] and has been implicated in contributing to inflammation [54, 57, 59]. Another possibility is that multiple cell types can be stained with the same cell markers; for example, megakaryocytes and platelets can be stained with CD31-specific antibody. Whether the virus actively replicates in these cells was not shown, and thus the dengue viral antigen detected in these cells may be the result of engulfed materials or undigested protein residue via in vivo deposition of virus-antibody complexes rather than direct infection. However, if stainings included a specific marker for platelets and/or megakaryocytes, it may help distinguish the phenotype of the dengue virus infected cells. Although these studies demonstrated that dengue viral antigens or RNA were observed in certain cell populations, the definitive phenotype was not determined. Therefore, in vivo, the cell(s) accounting for viremia during dengue virus infection remains an enigma.

## 9. Historical Observations

Retrospective literature reviews reveal that in bone marrows aspirated during the recovery stage or immediately after death, phagocytic clasmatocytes contain normoblastic, lymphocytic, granulocytic, erythrocytic, and platelet-like remnants in their cytoplasm [60–62]. Infected leukocytes (or monocytes) are frequently present on the last day, at the end of viremia, or the day after the disappearance of the virus from the plasma [63], suggesting that leukocytes may play an essential phagocytic role in the clearance of circulating virus. Recently, the phagocytic phenomenon has been confirmed in dengue hemorrhagic nonhuman primate model [64]. Due to difficulties and inconsistencies in identifying the cell lineages responsible for dengue viremia at the acute stage, monocytes and/or macrophages are gradually being assumed as the main cells for dengue virus propagation for the following reasons: (i) like the cells that can propagate the virus, they can adhere to cell culture flasks [63, 65], (ii) they are capable of phagocytosis [23, 66], and (iii) infrequently observed dengue viral antigens in cells with a similar morphology in tissues obtained postmortem [20, 67, 68]. These observations then led to the postulated hypothesis of antibody-dependent enhancement (ADE) [69] in an attempt to explain the epidemiological observation in which secondary infection with subsequent heterologous dengue serotypes is a risk factor for DHF/DSS [70]. The ADE theory is used to explain the severe dengue virus infection; antibody to the first infection may not be sufficient enough to neutralize a heterologous infection, and this partial cross-reacting antibody (or subneutralizing antibody) may promote Fc-bearing cells such as monocytes and macrophages to opsonize the virus, leading to increased virus production. 

However, studies have shown that some hematopoietic cells have the adherence and phagocytic property as well [71], and consequently reports on the ADE hypothesis are in debate. In support of this view, in the presence of subneutralizing antibody, a low percentage of dengue virus infected monocytes and/or macrophages can be observed in vitro [72–74]. On the contrary, some reports indicate that monocytes and/or macrophages have a different role—to protect against dengue virus replication. Evidences in support of this view include: (i) monocytes/macrophages undergo apoptosis in contact with dengue virus, (ii) they are capable of phagocytosis, (iii) they phagocytose infected apoptotic cells or apoptotic bodies, and (iv) they upregulate immune responses through autocrine or paracrine cytokine mechanisms [15, 64, 75–80]. 

An interesting discrepancy abounds. If monocytes and/or macrophages are the cells accounting for viremia during acute infection, why is it so difficult to detect the viral antigens in peripheral blood cells obtained from acute dengue patients? The aforementioned scenario—protective against dengue virus may account for the answer. With the evidence available in vivo to date, it is more reasonable to assume that the presence of dengue viral antigens within monocytes in samples obtained towards the end of the acute infection period may be the result of phagocytosis and viral clearance. Interestingly, a recent report also suggests a prominent role of monocytes and/or macrophages in the control of dengue virus in infected mice [81]. Unfortunately, the role of monocytes and/or macrophages in dengue virus infection has drawn the center attention for more than three decades, yet the importance they play in the pathogenesis of DHF/DSS is still unclear. Recently, an immunocompetent nonhuman primate model recapitulating the dengue hemorrhagic is available [64], the mystified issue on the role of monocytes and/or macrophages in dengue virus infection may be further delineated and hopefully resolved.

## 10. Biological Characteristics in Cells Infected by Dengue Virus

The reason why dengue viruses are capable of infecting a wide range of immortalized cell lines, such as myeloid-originated, B, T, fibroblast, and endothelial cells but yet comparatively poor at replicating in primary cells is currently unknown. Perhaps, it is likely that cell factors that are altered in immortalized cell lines contribute to this differential permissiveness. Immortalized cell lines are normally transformed with viruses, such as SV40 or EBV, which encode viral gene products that have an effect on interferon-signaling. Interestingly, among the cell mediator repertoire, interferon expression appears to be a very crucial element limiting the propagation of dengue virus [14, 82, 83]. In addition, defects in interferon signaling pathway has been shown in cancer cells, such as lymphoma and leukemia and established immortalized cell lines [84–88]. This line of evidence may, to some extent, explain why cell lines, such as Vero and K562 cells, which lack a functional interferon system, are highly permissive to dengue virus infection. In addition, activation of interferon-stimulated genes are the constant findings in cells with relatively poor permissive for dengue virus [14, 89, 90] and in specimens obtained in dengue-virus-infected humans and rhesus monkeys [89, 91, 92]. Within the same content, it is interesting to review what has been investigated in paucity of dengue animal models.

## 11. In Vivo Animal Studies

Currently, no perfect animal model that recapitulates the cardinal features of human DHF/DSS is available, even though a recent dengue hemorrhagic monkey model appears to be promising for dengue hemorrhagic investigation [64]. Since understanding the mechanisms leading to viremia and disease is necessary for vaccine and antiviral drug development, efforts have been made to search and/or generate a suitable dengue animal model. The readers should refer to recent review articles on the subject in smaller animals [93, 94]. This paper focuses mainly on why dengue viremia is seen in these animal models. 

The absence of disease symptoms, virus replication, and viremia in the serum of laboratory immunocompetent mice strains [95–98] indicates these mice are not suitable to study the cells permissive for dengue virus infection. In contrast, in immunocompromised mice, such as AG129, A/J, and STAT^−/−^mice [99–102], dengue viremia can be observed, though to some levels, in serum and in almost all the major organs studied. Thus, in immunocompromised mice, the interferon system may have defects that enhance disease unnaturally. Taking this into account, it is improbable that identification of the potential permissive cells for dengue virus replication will result from investigations with this model. In studies involving human chimeric mice, dengue virus appears to be detected predominantly in the human implanted or immortalized cells [103–109], suggesting that only the cells of human origin are infected and mice tissue can not support viremia. Nevertheless, as a whole, despite having a few drawbacks, such as low to undetectable dengue antibody in serum, and to some extent, lack of typical characteristics of dengue disease [108], currently a small animal model with detectable viremia, perhaps would be ideal for the initial screening of antiviral compounds and/or vaccine toxicity studies. However, the rhesus macaque animal model is more appropriate for investigations involving the cells responsible for dengue viremia. 

The only large animal species besides humans that are known to be naturally infected and can be experimentally infected by the parenteral route are monkeys [110–115] and apes [97]. The antibody response and viremia levels in monkeys are similar to that seen in humans [111], and therefore they have been viewed as an acceptable animal model to study virological and immunological aspects in experimental dengue virus infections [116–119]. In addition, it has been well documented that in all aspects, the cell composition of rhesus macaque bone marrow is very similar to that of humans [120, 121] and is highlighted by the fact that the parameters established for blood transfusions in monkeys has served as an important guide for these procedures in clinical studies [122]. Furthermore, a recent report demonstrated a recapitulation of human dengue hemorrhagic in rhesus monkeys via intravenous administration of high doses of dengue virus [64]. Even though the level and magnitude of dengue viremia is slightly lower than that of humans, this model displayed disease symptoms and thus is a better animal to investigate the source of dengue viremia. However, a systematic investigation to identify the potential cells for dengue viremia in this model has not been explored in depth due to limited accessibility of the resources and the high cost of the model. Thus, this topic will be evaluated with samples collected from dengue-infected patients.

## 12. In Vivo Dengue Patients

Studies over the years with specimens collected from the peripheral blood of dengue patients reveal that virus can be recovered or detected in a variety of cells. However, a general consensus concerning which cell lineages are involved in dengue viremia has never been conclusive, partly due to the variation of timing in specimen collection. Upon admission to the hospital with clinical symptoms, patients are always several days after the infection and frequently at the peak or downturn in viremia. By that time, a complex network of immune responses initiated and is in the action of viral clearance. Perhaps, this may explain why immune cells are commonly associated with the detection and/or isolation of virus in dengue patients [24]. Thus, the cells that are infected early, before the peak in viremia, and accounting for dengue viremia are still unknown.

### 12.1. Platelets in Dengue

One of the important clinical hallmarks in dengue virus infection in patients is platelet dysfunction, which occurs throughout the acute phase, and/or thrombocytopenia, which frequently occurs at the defebrile stage, thus this is a subject of interest, especially in understanding the possible mechanisms leading to the observed phenomena. There are a few proposed mechanisms that may explain platelet dysfunction and/or thrombocytopenia: (i) decreased production, (ii) direct infection by virus, (iii) increased consumption, or (iv) immune-complex lysis. The first mechanism has been observed. Early in infection of dengue virus, it exerts a transient depressive effect on megakaryocytes in the bone marrow [123–126], which subsequently becomes normocellular or hypercellular a few days after onset of fever [61, 124, 126]. In vitro and in vivo, dengue virus has been demonstrated to have toxic effects on platelets in the presence and absence of acute and convalescent patient serum, lending some support for the second mechanism [127–129]. In addition, dengue viral RNA has been isolated from or detected in platelets isolated from secondary dengue virus infected patients [130]. However, the precise mechanisms for the development of dysfunctional platelets and thrombocytopenia in dengue patients remain unknown. Also, the interactions of dengue virus with platelets, including entry and possible virus production, have not been investigated.

**Figure 2:**
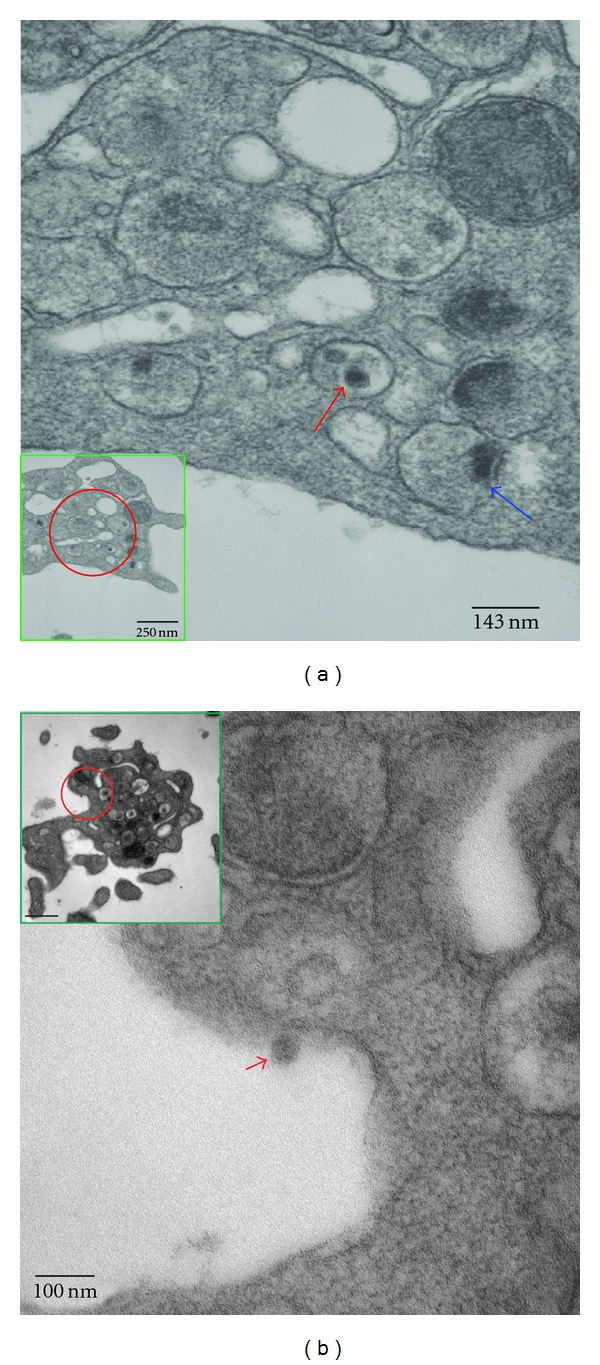
*Dengue virus-like particles in platelets isolated from confirmed dengue patients*. Platelets were isolated from confirmed dengue patients at the acute stage and subjected to electron microscopy. (a) Dengue virus-like particles were observed inside vesicle compartment (red arrow) and a particle appeared to be on its way budding out into the vesicle (blue arrow). (b) A single fuzzy virus-like particle was released from platelet (red arrow head). Red circle indicates the enlarged area. Insert is the platelets.

**Figure 3:**
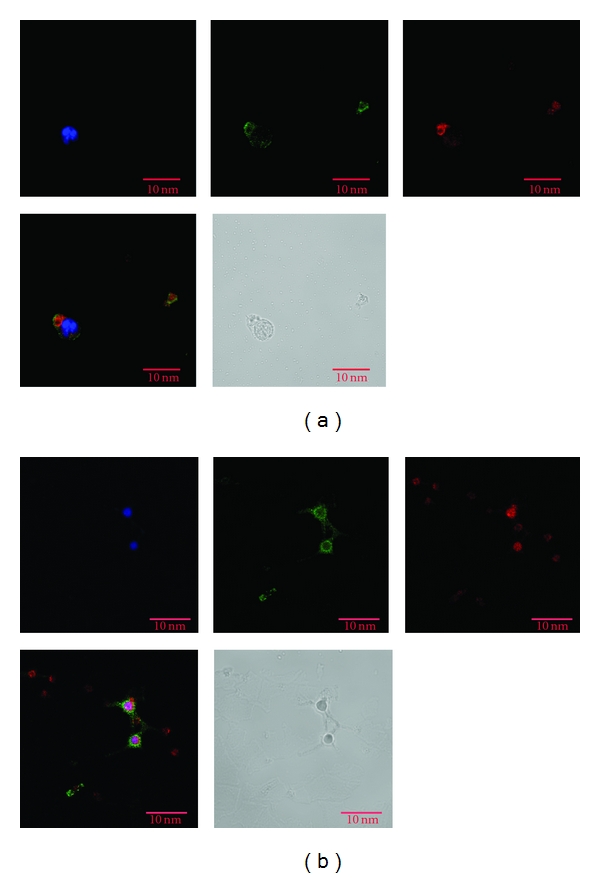
*Dengue antigens on platelets and its derivative cells*. Isolated platelets were stained with dengue-specific antibody (3H5) and platelets-specific markers (CD41). (a) Dengue antigen was observed in platelets and proplatelets. (b) Dengue antigen was observed in a micromegakaryocyte. Green: platelet marker CD41; Red: dengue antigen; and Blue; DAPI for nucleus staining. Red bar, 10 *μ*m.

We have proposed that platelets may be a critical element in early dengue virus infection [131–133], which may partially account for the dysfunction of platelets. Subsequent systematic investigations, with biological assays and electron microscopy, reveal that dengue viral RNA, either the positive stranded genome or negative stranded template, and the presence of mature virus-like particles, are consistently observed in platelets isolated from dengue confirmed patients during the acute phase of infection [132, 133]. A micrograph of dengue virus-like particles within platelets isolated from confirmed dengue patients is depicted (Figure 2). Typical clustering of dengue virus-like particles surrounded by a vesicle was observed in platelets (Figure 2(a)), and occasionally single or isolated dengue virus-like particles were observed [133]. Infrequently, dengue virus-like particle with a fuzzy morphology were observed associated with or released from platelets (Figure 2(b)). However, we could not rule out the possibility that these dengue virus-like particles containing platelets are in the category of megakaryocyte-derived microparticles [134]. In addition, immunofluorescent staining of platelets isolated from confirmed dengue patients reveals that viral antigens can be observed not only in platelets, but also in cells with the similar morphology as proplatelets (Figure 3(a)), while some dengue viral antigens were observed in presumably the micromegakaryocytes (Figure 3(b)). This observation is consistent with early reports by Nelson et al. [61, 126], who originally observed the presence of immature and nonplatelet forming megakaryocytes circulating in dengue patients and by Bhamarapravati and Boonyapaknavik [135], who noted that positive staining for dengue viral antigen in human tissues was demonstrated only in the lymphoid-like cells. Interestingly, the nucleated micromegakaryocytes, which are similar in size and morphology to lymphocytes, have been well documented [136, 137]. The presence of micromegakaryocytes, as opposed to megakaryocytes, suggests that production of platelets from bone marrow increases in response to dysfunctional or low numbers of platelets in the circulation of acute dengue patients.

**Figure 4:**
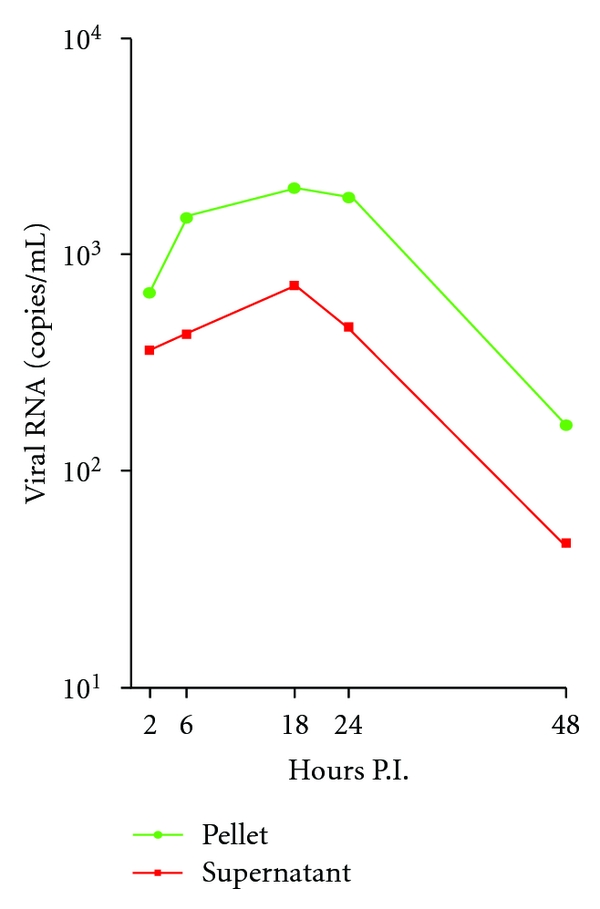
*Transient replication of dengue virus in platelets*. Platelets were isolated from a healthy donor and experimentally infected with dengue virus serotype 2 (strain 16681) at an MOI of 0.01. RNA was isolated from supernatants and pellets at indicated time and subjected to real-time qRT-PCR for dengue viral RNA.

Although platelets do not have a nucleus, they possess functional spliceosomes that are able to process pre-mRNAs into mature mRNA, from which proteins can be translated and processed [138, 139]. In vitro experiments were set up to investigate the susceptibility of platelets to support dengue virus production, which may directly contribute to the platelet dysfunction. A low level of dengue virus production could occur in infected platelets with the peak occurring at 18 hours post infection (Figure 4), suggesting that dengue virus is capable of replicating in platelets and dengue viral antigens may be expressed on the surface of platelets. Alternatively, the moderate viremia changes may result from the transient ability of platelets reproduction in culture conditions [140], which may have the capacity of capturing and releasing dengue viruses in later hours. Perhaps, this may account for the rise of platelet-associated antibodies (PAIgM/IgG) during acute dengue virus infection [130] and the increased incidence of phagocytosis of platelets from patients with secondary infections by human macrophages [78]. In addition, administration of intravenous immunoglobulin, which saturates phagocytosis and impedes antibody production, lacked efficacy when used to treat severe thrombocytopenic patients with secondary dengue virus infection [141]. As a whole, these evidences suggest that dengue virus may take a ride and experience ongoing maturation within platelets produced from infected progenitor megakaryocytes. 

Platelets are anucleate cells that have hemostatic and inflammatory functions [142, 143] and are composed of a concentrate of megakaryocyte membrane, cytoplasm, granules, and organelles [144]. Platelets circulate throughout blood vessels during which they monitor the integrity of the vascular system. All functional platelet responses must be tightly regulated to ensure that the formation of blood clots is of sufficient size to seal off the damaged area, while not disrupting blood flow to vital organs by causing vessel occlusion [145–147]. With the observation that dengue viral antigens are associated with proplatelets [148, 149] or micromegakaryocytes [137, 150] in blood during acute dengue virus infection (Figure 3), it is likely that a platelet lineage parental cell, megakaryocytes, may be involved in the production of dengue virus during acute infection. In addition, platelets contain several key elements related to dengue virus infection, such as DC-SIGN [151] as well as complement and Fc receptor, which have been implicated in virus uptake [152, 153]. It is also possible that a unique receptor or coreceptor is required for viral binding and entry into platelets. However, this particular receptor or coreceptor may not be evenly distributed or allocated in platelets since platelets are demarcated from the membrane of megakaryocytes, which may result in heterogeneous populations of platelets. This heterogeneity of platelet alloantigen referred to as human platelet alloantigen (HPA) polymorphism in the literature, and how it contributes to dengue virus infection and dengue disease severity warrants further investigation.

### 12.2. Megakaryocyte-Erythroid Progenitor (MEP) Cells in Dengue

Hematopoietic progenitor cells (HPCs) normally reside in bone marrow but can be mobilized to peripheral blood by stimulation with cytokines/chemokines. During infection, the microenvironment within the circulation contains a variety of immune cytokines/chemokines. Some of these immune cytokines/chemokines have the capacity to mobilize HPC to peripheral blood in response to the invading pathogen. CD41^+^CD61^+^ cells, such as megakaryocytes, normally account for 1% of the bone marrow but can change dramatically in certain diseases or infections and mobilize into the peripheral circulation. However, the presence of megakaryocytes in blood is a normal physiological occurrence [154]. Transport of megakaryocytes in the blood is halted in the lungs, where the majority shed their cytoplasm. Upon maturation via differentiation, the process of releasing platelets is initiated. Cytokines, such as thrombopoietin, can orchestrate the formation of platelets, which are held within the internal membranes in the cytoplasm of megakaryocytes. Platelets are released via two proposed scenarios [155]; (a) megakaryocytes undergo apoptosis to break up the platelets from demarcation of membranes, and (b) formation of platelet pseudopodia ribbons (proplatelets), which are released into blood vessels resulting in continuously release of platelets into the circulation. In either scenario, each megakaryocyte can give rise to 1000–3000 platelets [155], of which 2/3 of newly produced platelets remain in circulation while 1/3 is sequestered within the spleen. The remaining cell nucleus of the megakaryocyte, which is covered with a very thin cytoplasmic membrane and is morphologically similar to the small lymphocytes [137, 156], then crosses the bone marrow barrier into the blood and is consumed in the lung by macrophage-mediated phagocytosis [156]. 

Recently, a study profiling the gene expression by genome-scale transcriptional analysis in human primary megakaryocytic cell reveals that interferon-response genes are not induced or responsive to culture conditions or PMA treatment [157, 158], suggesting that there is a possible signaling defect in or impairment of interferon signaling in megakaryocytes. Thus, bone marrow suppression observed in dengue patients during the acute stage of infection, including reduction of megakaryocytes [61, 123, 125], may be due to direct disturbance or infection by dengue virus. Interestingly, damaged or degenerated megakaryocytes with homogenous hyalinized or reduced cytoplasms in bone marrow biopsies from acute patients have been documented [61, 123, 135, 159, 160]. Additionally, autopsies performed in patients who died of acute dengue hemorrhagic fever in the early 1960s revealed an increase in the number of megakaryocytes in the capillaries of various organs [161] and the deposition of hyaline materials with large mononuclear cells of varying maturity in the germinal centers of the spleen [162]. 

Furthermore, a unique and previously neglected cell population, which has ultrastructural and morphological appearance similar to that of micromegakaryocytes [137, 150] and a possible source of dengue viremia [22, 123], were seen in circulation during the acute phase of infection [129, 160], though the likely phenotypes of these neglected cells are not well defined. 

In addition, bone marrow aspiration studies show that erythroid cells are diminished transiently in all cases of dengue, some with an arrest of maturation [124, 126]. However, due to the long half-life of red blood cells in circulation, the transiently halted erythropoiesis does not cause severe anemia in dengue patients. This line of evidence suggests a possibility of a transient involvement of megakaryocyte-erythroid progenitor (MEP) cells in dengue virus infection. Whether direct infection of MEP cells or megakaryocytes by dengue virus can induce an aberrational transcriptional event, such as a disturbance of nucleic acid synthesis, resulting in the transiently halted erythropoiesis or increased production of immature megakaryocytes and atypical lymphocytes circulating in the blood remains unclear and warrants more exploration.

While dengue virus or its antigens has been found in several tissues and cells [15, 18, 20, 67, 162] from postmortem autopsy specimens and much important information has been generated; one thing has to be kept mind. By the time most of the patients are ill enough to be hospitalized, they are at the end stage of the dengue virus infection, multiple organ lesions or failures have occurred, and the virus or viral antigens may be trapped in these tissues and/or engulfed by phagocytic cells. Furthermore, a large number of macrophages containing what appears to be incompletely digested nuclear debris can be observed in autopsy specimens [62, 67, 161, 162], while the endothelial cells of the blood vessels look normal [67, 161, 162]. In addition, since dengue virus causes viremia in infected patients and the timing of the autopsy specimen collections are very critical, interpretation of outcomes may be complicated by the constant blood circulation in the body system when the patients are in consciousness. As a whole, at present, it is impossible to decipher the actual meaning of viral antigens or RNA in cells observed in autopsy specimens. With the recent suitable animal model, which is capable of recapitulating human dengue hemorrhages [64], the status of these cells may be clarified in the near future.

In summary, although many cell types including those paired with ADE capacity may play a role in dengue virus infection and in the development of DHF/DSS, this paper by no means suggests that cells with an impaired interferon system are the cells accounting for dengue viremia in vivo. Instead, this current paper addresses the observed phenomena in the literature and summarizes the possible scenarios. In addition, a new cell is suggested to have a role in DHF/DSS pathogenesis and warrants further investigation.

## 13. Conclusions

A new lineage of cell—MEP or CD41^+^CD61^+^ cells, such as megakaryocytes and/or platelets—is suggested for a potential cell accounting for dengue viremia in vivo. The objective of the authors is to draw scientific attention to the highly fragile cell with unusual biological properties in acute dengue virus infection. After all, hemostatic defects in DHF appear to be a major clinical finding. Our aim is to foster more detailed investigations of the MEP or CD41^+^CD61^+^ cells in specimens collected from acute dengue patients, which conceivably will not only provide a piece of valuable information of the mechanisms associated with DHF/DSS, but will also pave a new way on the formulation of effective candidate vaccines or antiviral drugs development.

## References

[1] Clark G, Gubler D, Dengue Fever J *CDC Traveler’s Information on Dengue Fever*.

[2] Guzmán MG, Kourí G, Díaz M (2004). Dengue, one of the great emerging health challenges of the 21st century. *Expert Review of Vaccines*.

[3] WHO (1999). *Prevention and Control of Dengue and Dengue Haemorrhagic Fever*.

[4] Halstead SB (2007). Dengue. *The Lancet*.

[5] NIAID

[6] Morens DM, Fauci AS (2008). Dengue and hemorrhagic fever: a potential threat to public health in the United States. *Journal of the American Medical Association*.

[7] Chambers TJ, Hahn CS, Galler R, Rice CM (1990). Flavivirus genome organization, expression, and replication. *Annual Review of Microbiology*.

[8] Sangkawibha N, Rojanasuphot S, Ahandrik S (1984). Risk factors in dengue shock syndrome: a prospective epidemiologic study in Rayong, Thailand. I. The 1980 outbreak. *American Journal of Epidemiology*.

[9] Andrews BS, Theofilopoulos AN, Peters CJ (1978). Replication of dengue and junin viruses in cultured rabbit and human endothelial cells. *Infection and Immunity*.

[10] Arévalo MT, Simpson-Haidaris PJ, Kou Z, Schlesinger JJ, Jin X (2009). Primary human endothelial cells support direct but not antibody-dependent enhancement of dengue viral infection. *Journal of Medical Virology*.

[11] Azizan A, Fitzpatrick K, Signorovitz A (2009). Profile of time-dependent VEGF upregulation in human pulmonary endothelial cells, HPMEC-ST1.6R infected with DENV-1, -2, -3, and -4 viruses. *Virology Journal*.

[12] Cabello-Gutiérrez C, Manjarrez-Zavala ME, Huerta-Zepeda A (2009). Modification of the cytoprotective protein C pathway during Dengue virus infection of human endothelial vascular cells. *Thrombosis and Haemostasis*.

[13] Kurane I, Hebblewaite D, Brandt WE, Ennis FA (1984). Lysis of dengue virus-infected cells by natural cell-mediated cytotoxicity and antibody-dependent cell-mediated cytotoxicity. *Journal of Virology*.

[14] Kurane I, Ennis FA (1988). Production of interferon alpha by dengue virus-infected human monocytes. *Journal of General Virology*.

[15] Kurane I, Kontny U, Janus J, Ennis FA (1990). Dengue-2 virus infection of human mononuclear cell lines and establishment of persistent infections. *Archives of Virology*.

[16] Sriurairatna S, Bhamarapravati N, Diwan AR, Halstead SB (1978). Ultrastructural studies on dengue virus infection of human lymphoblasts. *Infection and Immunity*.

[17] Theofilopoulos AN, Brandt WE, Russell PK, Dixon FT (1976). Replication of dengue 2 virus in cultured human lymphoblastoid cells and subpopulations of human peripheral leukocytes. *Journal of Immunology*.

[18] Balsitis SJ, Coloma J, Castro G (2009). Tropism of dengue virus in mice and humans defined by viral nonstructural protein 3-specific immunostaining. *The American Journal of Tropical Medicine & Hygiene*.

[19] Durbin AP, Vargas MJ, Wanionek K (2008). Phenotyping of peripheral blood mononuclear cells during acute dengue illness demonstrates infection and increased activation of monocytes in severe cases compared to classic dengue fever. *Virology*.

[20] Jessie K, Fong MY, Devi S, Lam SK, Wong KT (2004). Localization of dengue virus in naturally infected human tissues, by immunohistochemistry and in situ hybridization. *Journal of Infectious Diseases*.

[21] Zellweger RM, Prestwood TR, Shresta S (2010). Enhanced infection of liver sinusoidal endothelial cells in a mouse model of antibody-induced severe dengue disease. *Cell Host and Microbe*.

[22] Scott RM, Nisalak A, Cheamudon U (1980). Isolation of dengue viruses from peripheral blood leukocytes of patients with hemorrhagic fever. *Journal of Infectious Diseases*.

[23] Halstead SB, O’Rourke EJ, Allison AC (1977). Dengue viruses and mononuclear phagocytes. II. Identity of blood and tissue leukocytes supporting in vitro infection. *Journal of Experimental Medicine*.

[24] King AD, Nisalak A, Kalayanrooj S (1999). B cells are the principal circulating mononuclear cells infected by dengue virus. *Southeast Asian Journal of Tropical Medicine and Public Health*.

[25] Scott R, Nisalak A, Cheam-u-Dom U (1978). A preliminary report on the isolation of viruses from the platelets and leukocytes of dengue patients. *Asian Journal of Infectious Diseases*.

[26] Wu S-JL, Grouard-Vogel G, Sun W (2000). Human skin Langerhans cells are targets of dengue virus infection. *Nature Medicine*.

[27] Kyle JL, Beatty PR, Harris E (2007). Dengue virus infects macrophages and dendritic cells in a mouse model of infection. *Journal of Infectious Diseases*.

[28] Marovich M, Grouard-Vogel G, Louder M (2001). Human dendritic cells as targets of dengue virus infection. *Journal of Investigative Dermatology Symposium Proceedings*.

[29] Miller JL, DeWet BJM, Martinez-Pomares L (2008). The mannose receptor mediates dengue virus infection of macrophages. *PLoS Pathogens*.

[30] Nightingale ZD, Patkar C, Rothman AL (2008). Viral replication and paracrine effects result in distinct, functional responses of dendritic cells following infection with dengue 2 virus. *Journal of Leukocyte Biology*.

[31] Sun P, Fernandez S, Marovich MA (2009). Functional characterization of ex vivo blood myeloid and plasmacytoid dendritic cells after infection with dengue virus. *Virology*.

[32] Tassaneetrithep B, Burgess TH, Granelli-Piperno A (2003). DC-SIGN (CD209) mediates dengue virus infection of human dendritic cells. *Journal of Experimental Medicine*.

[33] Wang JP, Liu P, Latz E, Golenbock DT, Finberg RW, Libraty DH (2006). Flavivirus activation of plasmacytoid dendritic cells delineates key elements of TLR7 signaling beyond endosomal recognition. *Journal of Immunology*.

[34] Kwan W-H, Navarro-Sanchez E, Dumortier H (2008). Dermal-type macrophages expressing CD209/DC-SIGN show inherent resistance to dengue virus growth. *PLoS Neglected Tropical Diseases*.

[35] Limon-Flores AY, Perez-Tapia M, Estrada-Garcia I (2005). Dengue virus inoculation to human skin explants: an effective approach to assess in situ the early infection and the effects on cutaneous dendritic cells. *International Journal of Experimental Pathology*.

[36] Taweechaisupapong S, Sriurairatana S, Angsubhakorn S, Yoksan S, Bhamarapravati N (1996). In vivo and in vitro studies on the morphological change in the monkey epidermal Langerhans cells following exposure to dengue 2 (16681) virus. *Southeast Asian Journal of Tropical Medicine and Public Health*.

[37] Jampangern W, Vongthoung K, Jittmittraphap A (2007). Characterization of atypical lymphocytes and immunophenotypes of lymphocytes in patients with dengue virus infection. *Asian Pacific Journal of Allergy and Immunology*.

[38] Boonpucknavig S, Lohachitranond C, Nimmanitya S (1979). The pattern and nature of the lymphocyte population response in dengue hemorrhagic fever. *The American Journal of Tropical Medicine & Hygiene*.

[39] Wells RA, Scott McN. R, Pavanand K (1980). Kinetics of peripheral blood leukocyte alterations in Thai children with dengue hemorrhagic fever. *Infection and Immunity*.

[40] O’Neill LAJ (2003). Therapeutic targeting of Toll-like receptors for inflammatory and infectious diseases. *Current Opinion in Pharmacology*.

[41] Bechetoille N, André V, Valladeau J, Perrier E, Dezutter-Dambuyant C (2006). Mixed Langerhans cell and interstitial/dermal dendritic cell subsets emanating from monocytes in Th2-mediated inflammatory conditions respond differently to proinflammatory stimuli. *Journal of Leukocyte Biology*.

[42] De Witte L, Nabatov A, Pion M (2007). Langerin is a natural barrier to HIV-1 transmission by Langerhans cells. *Nature Medicine*.

[43] de Witte L, Nabatov A, Geijtenbeek TBH (2008). Distinct roles for DC-SIGN+-dendritic cells and Langerhans cells in HIV-1 transmission. *Trends in Molecular Medicine*.

[44] Nagao K, Ginhoux F, Leitner WW (2009). Murine epidermal Langerhans cells and langerin-expressing dermal dendritic cells are unrelated and exhibit distinct functions. *Proceedings of the National Academy of Sciences of the United States of America*.

[45] Gordon RM, Lumsden WHR (1939). A study of the behaviour of the mouth-parts of mosquitoes when taking up blood from living tissues; together with some observations on the ingestion of microfilariae. *Annals of Tropical Medicine and Parasitology*.

[46] Griffiths RB, Gordon RM (1952). An apparatus which enables the process of feeding by mosquitoes to be observed in the tissues of a live rodent; together with an account of the ejection of saliva and its significance in Malaria. *Annals of Tropical Medicine and Parasitology*.

[47] O’Rourke FJ (1956). Observations on pool and capillary feeding in aedes aegypt. *Nature*.

[48] Daniel TL, Kingsolver JG (1983). Feeding strategy and the mechanics of blood sucking in insects. *Journal of Theoretical Biology*.

[49] Ramasubramanian MK, Barham OM, Swaminathan V (2008). Mechanics of a mosquito bite with applications to microneedle design. *Bioinspiration and Biomimetics*.

[50] Styer LM, Kent KA, Albright RG, Bennett CJ, Kramer LD, Bernard KA (2007). Mosquitoes inoculate high doses of West Nile virus as they probe and feed on live hosts. *PLoS Pathogens*.

[51] Davis MM (2008). A Prescription for Human Immunology. *Immunity*.

[52] Mestas J, Hughes CCW (2004). Of Mice and Not Men: differences between mouse and human immunology. *Journal of Immunology*.

[53] Yang K, Puel A, Zhang S (2005). Human TLR-7-, -8-, and -9-mediated induction of IFN-alpha/beta and -lambda Is IRAK-4 dependent and redundant for protective immunity to viruses. *Immunity*.

[54] Boilard E, Nigrovic PA, Larabee K (2010). Platelets amplify inflammation in arthritis via collagen-dependent microparticle production. *Science*.

[55] Faint RW (1992). Platelet-neutrophil interactions: their significance. *Blood Reviews*.

[56] Gawaz MP, Mujais SK, Schmidt B, Gurland HJ (1994). Platelet-leukocyte aggregation during hemodialysis. *Kidney International*.

[57] Reuter S, Lang D (2009). Life span of monocytes and platelets: importance of interactions. *Frontiers in Bioscience*.

[58] Rinder HM, Bonan JL, Rinder CS, Ault KA, Smith BR (1991). Activated and unactivated platelet adhesion to monocytes and neutrophils. *Blood*.

[59] Bazzoni G, Dejana E, Del Maschio A (1991). Platelet-neutrophil interactions. Possible relevance in the pathogenesis of thrombosis and inflammation. *Haematologica*.

[60] Aikat BK (1966). Pathology of mosquito-borne haemorrhagic fever in the Calcutta outbreak. *Bull World Health Organ*.

[61] Nelson ER, Bierman HR, Chulajata R (1964). Hematologic findings in the 1960 hemorrhagic fever epidemic (dengue) in Thailand. *The American Journal of Tropical Medicine & Hygiene*.

[62] Nelson ER, Bierman HR, Chulajata R (1966). Hematologic phagocytosis in postmortem bone marrows of dengue hemorrhagic fever. (Hematologic phagocytosis in Thai hemorrhagic fever). *American Journal of the Medical Sciences*.

[63] Marchette NJ, Halstead SB, Falkler WA (1973). Studies on the pathogenesis of dengue infection in monkeys. III. Sequential distribution of virus in primary and heterologous infections. *Journal of Infectious Diseases*.

[64] Onlamoon N, Noisakran S, Hsiao H-M (2010). Dengue virus—induced hemorrhage in a nonhuman primate model. *Blood*.

[65] Marchette NJ, Sung Chow JS, Halstead SB (1975). Dengue virus replication in cultures of peripheral blood leukocytes during the course of Dengue haemorrhagic fever. *Southeast Asian Journal of Tropical Medicine and Public Health*.

[66] Marchette NJ, Halstead SB, Chow JS (1976). Replication of dengue viruses in cultures of peripheral blood leukocytes from dengue immune rhesus monkeys. *Journal of Infectious Diseases*.

[67] Bhamarapravati N, Tuchinda P, Boonyapaknavik V (1967). Pathology of Thailand haemorrhagic fever: a study of 100 autopsy cases. *Annals of Tropical Medicine and Parasitology*.

[68] de Macedo FC, Nicol AF, Cooper LD, Yearsley M, Cordovil Pires AR, Nuovo GJ (2006). Histologic, viral, and molecular correlates of dengue fever infection of the liver using highly sensitive immunohistochemistry. *Diagnostic Molecular Pathology*.

[69] Halstead SB, O’Rourke EJ (1977). Antibody enhanced dengue virus infection in primate leukocytes. *Nature*.

[70] Halstead SB (1970). Observations related to pathogensis of dengue hemorrhagic fever. VI. Hypotheses and discussion. *Yale Journal of Biology and Medicine*.

[71] Eaves AC, Cashman JD, Gaboury LA (1986). Unregulated proliferation of primitive chronic myeloid leukemia progenitors in the presence of normal marrow adherent cells. *Proceedings of the National Academy of Sciences of the United States of America*.

[72] Blackley S, Kou Z, Chen H (2007). Primary Human splenic macrophages, but not T or B cells, are the principal target cells for dengue virus infection in vitro. *Journal of Virology*.

[73] Huang K-J, Yang Y-C, Lin Y-S (2006). The dual-specific binding of dengue virus and target cells for the antibody-dependent enhancement of dengue virus infection. *Journal of Immunology*.

[74] Kliks SC, Nisalak A, Brandt WE, Wahl L, Burke DS (1989). Antibody-dependent enhancement of dengue virus growth in human monocytes as a risk factor for dengue hemorrhagic fever. *The American Journal of Tropical Medicine & Hygiene*.

[75] Espina LM, Valero NJ, Hernández JM, Mosquera JA (2003). Increased apoptosis and expression of tumor necrosis factor-*α* caused by infection of cultured human monocytes with dengue virus. *The American Journal of Tropical Medicine & Hygiene*.

[76] Hase T, Summers PL, Eckels KH (1989). Flavivirus entry into cultured mosquito cells and human peripheral blood monocytes. *Archives of Virology*.

[77] Hober D, Nguyen TL, Shen L (1998). Tumor necrosis factor alpha levels in plasma and whole-blood culture in dengue-infected patients: relationship between virus detection and pre-existing specific antibodies. *Journal of Medical Virology*.

[78] Honda S, Saito M, Dimaano EM (2009). Increased phagocytosis of platelets from patients with secondary dengue virus infection by human macrophages. *The American Journal of Tropical Medicine & Hygiene*.

[79] Marianneau P, Steffan A-M, Royer C (1999). Infection of primary cultures of human Kupffer cells by dengue virus: no viral progeny synthesis, but cytokine production is evident. *Journal of Virology*.

[80] Mosquera JA, Hernandez JP, Valero N, Espina LM, Añez GJ (2005). Ultrastructural studies on dengue virus type 2 infection of cultured human monocytes. *Virology Journal*.

[81] Fink K, Ng C, Nkenfou C, Vasudevan SG, Van Rooijen N, Schul W (2009). Depletion of macrophages in mice results in higher dengue virus titers and highlights the role of macrophages for virus control. *European Journal of Immunology*.

[82] Calvert AE, Huang CY-H, Kinney RM, Roehrig JT (2006). Non-structural proteins of dengue 2 virus offer limited protection to interferon-deficient mice after dengue 2 virus challenge. *Journal of General Virology*.

[83] Diamond MS, Roberts TG, Edgil D, Lu B, Ernst J, Harris E (2000). Modulation of dengue virus infection in human cells by alpha, beta, and gamma interferons. *Journal of Virology*.

[84] Diaz MO, Ziemin S, Le Beau MM (1988). Homozygous deletion of the *α*- and *β*1-interferon genes in human leukemia and derived cell lines. *Proceedings of the National Academy of Sciences of the United States of America*.

[85] Diaz MO, Rubin CM, Harden A (1990). Deletions of interferon genes in acute lymphoblastic leukemia. *The New England Journal of Medicine*.

[86] Einhorn S, Grander D, Bjork O, Brondum-Nielsen K, Soderhall S (1990). Deletion of f alpha-, beta-, and omega-interferon genes in malignant cells from children with acute lymphocytic leukemia. *Cancer Research*.

[87] James CD, He J, Carlbom E, Nordenskjold M, Cavenee WK, Collins VP (1991). Chromosome 9 deletion mapping reveals interferon *α* and interferon *β*-1 gene deletions in human glial tumors. *Cancer Research*.

[88] Miyakoshi J, Dobler KD, Allalunis-Turner J (1990). Absence of IFNA and IFNB genes from human malignant glioma cell lines and lack of correlation with cellular sensitivity to interferons. *Cancer Research*.

[89] Fink J, Gu F, Ling L (2007). Host gene expression profiling of dengue virus infection in cell lines and patients. *PLoS Neglected Tropical Diseases*.

[90] Kurane I, Janus J, Ennis FA (1992). Dengue virus infection of human skin fibroblasts in vitro production of IFN-*β*, IL-6 and GM-CSF. *Archives of Virology*.

[91] Kurane I, Innis BL, Nimmannitya S, Nisalak A, Meager A, Ennis FA (1993). High levels of interferon alpha in the sera of children with dengue virus infection. *The American Journal of Tropical Medicine & Hygiene*.

[92] Sariol CA, Muñoz-Jordan JL, Abel K (2007). Transcriptional activation of interferon-stimulated genes but not of cytokine genes after primary infection of rhesus macaques with dengue virus type 1. *Clinical and Vaccine Immunology*.

[93] Bente DA, Rico-Hesse R (2006). Models of dengue virus infection. *Drug Discovery Today: Disease Models*.

[94] Yauch LE, Shresta S (2008). Mouse models of dengue virus infection and disease. *Antiviral Research*.

[95] Chen H-C, Lai S-Y, Sung J-M (2004). Lymphocyte activation and hepatic cellular infiltration in immunocompetent mice infected by dengue virus. *Journal of Medical Virology*.

[96] Paes MV, Pinhão AT, Barreto DF (2005). Liver injury and viremia in mice infected with dengue-2 virus. *Virology*.

[97] Paul JR, Melnick JL, Sabin AB (1948). Experimental attempts to transmit phlebotomus and dengue fevers to chimpanzees. *Proceedings of The Society for Experimental Biology and Medicine*.

[98] Sabin AB (1952). Research on dengue during World War II. *The American Journal of Tropical Medicine & Hygiene*.

[99] Chen S-T, Lin Y-L, Huang M-T (2008). CLEC5A is critical for dengue-virus-induced lethal disease. *Nature*.

[100] Huang Y-H, Lei H-Y, Liu H-S, Lin Y-S, Liu C-C, Yeh T-M (2000). Dengue virus infects human endothelial cells and induces IL-6 and IL-8 production. *The American Journal of Tropical Medicine & Hygiene*.

[101] Johnson AJ, Roehrig JT (1999). New mouse model for dengue virus vaccine testing. *Journal of Virology*.

[102] Shresta S, Sharar KL, Prigozhin DM, Beatty PR, Harris E (2006). Murine model for dengue virus-induced lethal disease with increased vascular permeability. *Journal of Virology*.

[103] An J, Kimura-Kuroda J, Hirabayashi Y, Yasui K (1999). Development of a novel mouse model for dengue virus infection. *Virology*.

[104] Bente DA, Melkus MW, Garcia JV, Rico-Hesse R (2005). Dengue fever in humanized NOD/SCID mice. *Journal of Virology*.

[105] Blaney JE, Johnson DH, Manipon GG (2002). Genetic basis of attenuation of dengue virus type 4 small plaque mutants with restricted replication in suckling mice and in SCID mice transplanted with human liver cells. *Virology*.

[106] Kuruvilla JG, Troyer RM, Devi S, Akkina R (2007). Dengue virus infection and immune response in humanized RAG2-/-*γ*c-/- (RAG-hu) mice. *Virology*.

[107] Lin Y-L, Liao C-L, Chen L-K (1998). Study of dengue virus infection in SCID mice engrafted with human K562 cells. *Journal of Virology*.

[108] Mota J, Rico-Hesse R (2009). Humanized mice show clinical signs of dengue fever according to infecting virus genotype. *Journal of Virology*.

[109] Wu S-JL, Hayes CG, Dubois DR (1995). Evaluation of the severe combined immunodeficient (SCID) mouse as an animal model for dengue viral infection. *The American Journal of Tropical Medicine & Hygiene*.

[110] Halstead SB (1979). In vivo enhancement of Dengue virus infection in rhesus monkeys by passively transferred antibody. *Journal of Infectious Diseases*.

[111] Marchette NJ, Halstead SB (1974). Immunopathogenesis of dengue infection in the rhesus monkey. *Transplantation Proceedings*.

[112] Rosen L (1958). Experimental infection of New World monkeys with dengue and yellow fever viruses. *The American Society of Tropical Medicine & Hygiene*.

[113] Rudnick A, Marchette NJ, Garcia R (1967). Possible jungle dengue—recent studies and hypotheses. *Japanese Journal of Medical Science and Biology*.

[114] Simmons JS, St. John JH, Reynolds FHK (1931). Experimental studies of dengue. *Philippine Journal of Science*.

[115] Whitehead RH, Chaicumpa V, Olson LC, Russell PK (1970). Sequential dengue virus infections in the white-handed gibbon (Hylobates lar). *The American Journal of Tropical Medicine & Hygiene*.

[116] Guirakhoo F, Pugachev K, Arroyo J (2002). Viremia and immunogenicity in nonhuman primates of a tetravalent yellow fever-dengue chimeric vaccine: genetic reconstructions, dose adjustment, and antibody responses against wild-type dengue virus isolates. *Virology*.

[117] Guy B, Barban V, Mantel N (2009). Evaluation of interferences between dengue vaccine serotypes in a monkey model. *The American Journal of Tropical Medicine & Hygiene*.

[118] Koraka P, Benton S, Amerongen GV, Stittelaar KJ, Osterhaus ADME (2007). Characterization of humoral and cellular immune responses in cynomolgus macaques upon primary and subsequent heterologous infections with dengue viruses. *Microbes and Infection*.

[119] Velzing J, Groen J, Drouet MT (1999). Induction of protective immunity against dengue virus type 2: comparison of candidate live attenuated and recombinant vaccines. *Vaccine*.

[120] Stasney J, Higgins GM (1973). Bone marrow in the monkey (macacus rhesus). *The Anatomical Record*.

[121] Wills L, Stewart A (1935). Experimental Anaemia in monkeys, with special reference to macrocytic nutritional anaemia. *British Journal of Experimental Pathology*.

[122] Kushida T, Inaba M, Ikebukuro K (2002). Comparison of bone marrow cells harvested from various bones of cynomolgus monkeys at various ages by perfusion or aspiration methods: a preclinical study for human BMT. *Stem Cells*.

[123] Bierman HR, Nelson ER (1965). Hematodepressive virus diseases of Thailand. *Annals of Internal Medicine*.

[124] Na-Nakorn S, Suingdumrong A, Pootrakul S, Bhamarapravati N (1966). Bone-marrow studies in Thai haemorrhagic fever. *Bull World Health Organ*.

[125] Nelson ER, Bierman HR (1964). Dengue fever: a thrombocytopenic disease?. *Journal of the American Medical Association*.

[126] Nelson ER, Tuchinda S, Bierman HR, Chulajata R (1966). Haematology of Thai haemorrhagic fever (dengue). *Bull World Health Organ*.

[127] Oishi K, Saito M, Mapua CA, Natividad FF (2007). Dengue illness: clinical features and pathogenesis. *Journal of Infection and Chemotherapy*.

[128] Schexneider KI, Reedy EA (2005). Thrombocytopenia in dengue fever. *Current Hematology Reports*.

[129] Srichaikul T, Nimmannitya S (2000). Haematology in dengue and dengue haemorrhagic fever. *Bailliere’s Best Practice and Research in Clinical Haematology*.

[130] Saito M, Oishi K, Inoue S (2004). Association of increased platelet-associated immunoglobulins with thrombocytopenia and the severity of disease in secondary dengue virus infections. *Clinical and Experimental Immunology*.

[131] Noisakran S, Guey CP (2008). Alternate hypothesis on the pathogenesis of dengue hemorrhagic fever (DHF)/dengue shock syndrome (DSS) in dengue virus infection. *Experimental Biology and Medicine*.

[132] Noisakran S, Chokephaibulkit K, Songprakhon P (2009). A re-evaluation of the mechanisms leading to dengue hemorrhagic fever. *Annals of the New York Academy of Sciences*.

[133] Noisakran S, Gibbons RV, Songprakhon P (2009). Detection of dengue virus in platelets isolated from dengue patients. *Southeast Asian Journal of Tropical Medicine and Public Health*.

[134] Flaumenhaft R, Dilks JR, Richardson J (2009). Megakaryocyte-derived microparticles: direct visualization and distinction from platelet-derived microparticles. *Blood*.

[135] Bhamarapravati N, Boonyapaknavik V, Nimsomburana P (1966). Pathology of Thai haemorrhagic fever: an autopsy study. *Bull World Health Organ*.

[136] Gorius JB, Dreyfus B, Sultan C, Basch A, d’Oliveira JG (1972). Identification of circulating micromegakaryocytes in a case of refractory anemia: an electron microscopic-cytochemical study. *Blood*.

[137] Yamauchi K, Miyauchi J, Nagao T (1984). Identification of circulating micromegakaryocytes in a case of erythroleukemia. *Cancer*.

[138] Denis MM, Tolley ND, Bunting M (2005). Escaping the nuclear confines: signal-dependent pre-mRNA splicing in anucleate platelets. *Cell*.

[139] Schwertz H, Tolley ND, Foulks JM (2006). Signal-dependent splicing of tissue factor pre-mRNA modulates the thrombogenecity of human platelets. *Journal of Experimental Medicine*.

[140] Schwertz H, Köster S, Kahr WH (2010). Anucleate platelets generate progeny. *Blood*.

[141] Dimaano EM, Saito M, Honda S (2007). Lack of efficacy of high-dose intravenous immunoglobulin treatment of severe thrombocytopenia in patients with secondary dengue virus infection. *The American Journal of Tropical Medicine & Hygiene*.

[142] Jurk K, Kehrel BE (2005). Platelets: physiology and biochemistry. *Seminars in Thrombosis and Hemostasis*.

[143] Ruggeri ZM (2002). Platelets in atherothrombosis. *Nature Medicine*.

[144] Hartwig JH (2006). The platelet: form and function. *Seminars in Hematology*.

[145] Andrews RK, Berndt MC (2004). Platelet physiology and thrombosis. *Thrombosis Research*.

[146] Kaushansky K (2008). Historical review: megakaryopoiesis and thrombopoiesis. *Blood*.

[147] Rendu F, Brohard-Bohn B (2001). The platelet release reaction: granules’ constituents, secretion and functions. *Platelets*.

[148] Italiano JE, Lecine P, Shivdasani RA, Hartwig JH (1999). Blood platelets are assembled principally at the ends of proplatelet processes produced by differentiated megakaryocytes. *Journal of Cell Biology*.

[149] Patel SR, Hartwig JH, Italiano JE (2005). The biogenesis of platelets from megakaryocyte proplatelets. *Journal of Clinical Investigation*.

[150] Erber WN, Jacobs A, Oscier DG, O’Hea AM, Mason DY (1987). Circulating micromegakaryocytes in myelodysplasia. *Journal of Clinical Pathology*.

[151] Boukour S, Massé J-M, Bénit L, Dubart-Kupperschmitt A, Cramer EM (2006). Lentivirus degradation and DC-SIGN expression by human platelets and megakaryocytes. *Journal of Thrombosis and Haemostasis*.

[152] Anderson CL, Chacko GW, Osborne JM, Brandt JT (1995). The Fc receptor for immunoglobulin G (Fc*γ*RII) on human platelets. *Seminars in Thrombosis and Hemostasis*.

[153] Skoglund C, Wetterö J, Skogh T, Sjöwall C, Tengvall P, Bengtsson T (2008). C-reactive protein and C1q regulate platelet adhesion and activation on adsorbed immunoglobulin G and albumin. *Immunology and Cell Biology*.

[154] Woods MJ, Greaves M, Trowbridge EA (1992). The physiological significance of circulating megakaryocytes. *British Journal of Haematology*.

[155] Stenberg PE, Levin J (1989). Mechanisms of platelet production. *Blood Cells*.

[156] Radley JM, Haller CJ (1983). Fate of senescent megakaryocytes in the bone marrow. *British Journal of Haematology*.

[157] Fuhrken PG, Chen C, Miller WM, Papoutsakis ET (2007). Comparative, genome-scale transcriptional analysis of CHRF-288-11 and primary human megakaryocytic cell cultures provides novel insights into lineage-specific differentiation. *Experimental Hematology*.

[158] Kim J-A, Jung Y-J, Seoh J-Y, Woo S-Y, Seo J-S, Kim H-L (2002). Gene expression profile of megakaryocytes from human cord blood CD34+ cells ex vivo expanded by thrombopoietin. *Stem Cells*.

[159] Bhamarapravati N, Halstead SB, Sookavachana P, Boonyapaknavik V (1964). Studies on dengue virus infection. 1. immunofluorescent localization of virus in mouse tissue. *Archives of Pathology*.

[160] Reyes VM (1966). The pathology of haemorrhagic fever in the Philippines. *Bull World Health Organ*.

[161] Bhamarapravati N (1961). The spectrum of pathological changes in Thai haemorrhagic fever. *SEATO Medical Research Monograph*.

[162] Piyaratn P (1961). Pathology of Thailand epidemic hemorrhagic fever. *The American Journal of Tropical Medicine & Hygiene*.

